# Supplementary material for: Efficiency of Lactiplantibacillus plantarum JT-PN39 and Paenibacillus motobuensis JT-A29 for Fermented Coffee Applications and Fermented Coffee Characteristics
Source: Foods. 2023 Jul 29;12(15):2894. doi: 10.3390/foods12152894 (PMC10418488; doi:10.3390/foods12152894)
Supplement: Supplementary file 1 [file foods-12-02894-s001.zip › foods-2466134-supplementary.pdf]

Table S1. Non-volatile compounds in roasted coffee examination by LC-MS.

Non-volatile	Molecular formula	MW (g/mol)	t_R(min)	Area x10^5					
				OC	C	PM	LP	PM+LP	CC
Trigonelline	$C_7H_7NO_2$	136.0404	8.40	4.130	7.160	5.530	**6.950**	5.210	5.920
Chlorogenic acid	$C_{16}H_{18}O_9$	353.0879	9.42	1,100.000	1,820.000	1,870.000	**2,230.000**	2,010.000	1,360.000
Ferulic acid	$C_{10}H_{10}O_4$	193.0505	11.93	6.690	7.790	7.240	**8.310**	5.560	7.880
Quinic acid	$C_7H_{12}O_6$	191.0561	1.38	2,280.000	2,280.000	1,870.000	2,010.000	1,780.000	**2,300.000**
Malic acid	$C_4H_6O_5$	133.0143	1.74	222.000	247.000	183.000	**276.000**	**272.000**	344.000
Lactic acid	$C_3H_6O_3$	89.02402	1.77	111.000	158.000	516.000	**1,830.000**	1,390.000	130.000
Citric acid	$C_6H_8O_7$	191.0198	1.92	-	16.000	0.558	53.400	31.900	**181.000**
Tartaric acid	$C_4H_6O_6$	149.0091	1.75	0.130	**0.262**	-	0.116	0.028	0.111
Oxalic acid	$C_2H_2O_4$	88.9876	1.74	**14.800**	7.960	12.300	12.200	12.700	13.700
Citramalic acid	$C_5H_8O_5$	147.0299	1.56	16.800	**43.900**	1.200	22.300	9.590	26.900
Glutamic acid	$C_5H_9NO_4$	146.0458	1.77	0.713	1.040	1.120	1.240	**1.680**	0.828
Succinic acid	$C_4H_6O_4$	117.0194	2.11	17.900	50.300	22.600	**182.000**	51.300	33.500
Sucrose	$C_{12}H_{22}O_{11}$	341.1089	1.39	38.100	36.700	19.600	29.900	19.000	**45.900**
Fructose	$C_6H_{12}O_6$	179.0561	1.55	**139.000**	45.500	93.800	50.900	41.900	108.000
Catechins	$C_{15}H_{14}O_6$	289.0719	10.79	3.660	8.820	5.720	**9.210**	5.800	6.840

Table S1. (continued)

Non-volatile	Molecular formula	MW (g/mol)	t_R(min)	Area x10^5					
				OC	C	PM	LP	PM+LP	CC
Quercetin	$C_{15}H_{10}O_7$	301.0354	1.46	0.106	**0.166**	0.127	0.165	0.304	-
1,3-Dicaffeoylquinic acid	$C_{25}H_{24}O_{12}$	515.1196	11.97	2.340	**11.900**	0.457	7.650	8.380	8.150
Niacin (Nicotinic acid)	$C_6H_5NO_2$	122.0247	1.34	12.100	12.700	**16.600**	15.900	14.500	11.100
Theophylline	$C_7H_8N_4O_2$	179.0575	7.02	0.123	0.564	0.339	**0.458**	0.319	0.341
Shikimic acid	$C_7H_{10}O_5$	173.0454	1.42	33.200	16.800	18.600	**43.100**	40.900	18.300
Beta-D-galacturonic acid	$C_6H_{10}O_7$	193.0351	1.41	2.360	9.280	13.600	14.100	**14.500**	5.240
3-Hydroxyanthranilic acid	$C_7H_7NO_3$	152.0353	9.29	21.300	42.600	71.200	54.400	**47.600**	23.200
Chorismate	$C_{10}H_{10}O_6$	225.0404	4.69	0.526	0.476	0.383	0.483	0.381	**0.680**
Galactomannan	$C_{18}H_{32}O_{16}$	503.1620	1.40	4.158	3.856	3.486	4.560	3.264	**5.110**
Cafeic acid pathway									
L-Tyrosine	$C_9H_{11}NO_3$	180.0667	11.84	2.290	4.940	3.080	5.710	4.990	**5.230**
N-Hydroxy-L-tyrosine	$C_9H_{11}NO_4$	197.0688	9.05	1.870	7.340	6.190	**12.500**	9.490	4.430
3-Coumaric acid	$C_9H_8O_3$	164.0473	11.13	3.880	4.830	**5.890**	5.100	5.660	4.120
Cafeic acid	$C_9H_8O_4$	180.16	9.84	26.500	64.000	57.600	63.700	**64.800**	44.100

Table S1. (continued)

Non-volatile	Molecular formula	MW (g/mol)	t_R(min)	Area x10^5					
				OC	C	PM	LP	PM+LP	CC
Caffeine pathway									
Xanthosine	C10H12N4O6	283.0672	1.42	0.119	**0.699**	0.201	1.130	0.622	0.445
7-Methylxanthosine	C11H15N4O6	298.0932	10.45	0.571	2.720	3.270	5.120	4.340	**0.955**
7-Methylxanthine	C6H6N4O2	165.0405	1.58	0.195	0.116	0.476	0.101	0.287	**0.684**
Theobromine	C7H8N4O2	179.0574	7.02	0.081	**0.564**	0.227	0.458	0.319	0.236
Caffeine	$C_8H_{10}N_4O_2$	193.0731	1.42	**1.700**	1.620	0.339	0.741	0.491	1.610

Note: ordinary coffee (OC), control or non-fermentation (C; uninoculation), *P. motobuensis* JT-A29 (PM), *L. plantarum* JT-PN39 (LP), mixed starter cultures (PM+LP) fermented coffee and Civet coffee (CC).

Table S2. Volatile compounds in roasted coffee beans

Compound	Aroma	Group	rt	Total peak area percentage (%)				
				OC	C	LP	PM+LP	PM
Acetic acid	sour	Acid	1.978	31.42	31.49	**59.92**	20.63	4.95
Butanoic acid	rancid butter, sour	Acid	2.824	0.00	0.00	0.00	**15.87**	0.00
**Furfural**	**almond, nutty, sweet, caramel, bread**	**Furans**	3.399	33.49	44.01	**68.37**	59.76	**64.14**
2-Furanmethanol	caramellic, burnt, smoky	Alcohol	3.667	**100.00**	**100.00**	78.23	**100.00**	**100.00**
Pyrazine, 2,5-dimethyl-	coffee, cocoa, nutty, roasted, grassy	Pyrazine	4.743	38.94	**63.17**	47.88	44.72	22.08
**5-Methyl furfural**	**sweet, caramel, coffee**	**Furans**	5.946	55.91	54.92	**100.00**	59.66	**66.13**
Pyrazine, 2-ethyl-6-methyl-	roasted, hazelnut-like	Pyrazine	6.907	0.00	**41.88**	0.00	34.76	0.00
3,5-Dimethylcyclohex-1-ene-4-carboxaldehyde	floral	Aldehyde	9.326	1.17	0.00	**2.07**	0.00	0.00
Pyrazine, 2-ethyl-3,5-dimethyl-	wheat bread, smoked fatty fish, roast chicken, roast beef, black tea	Pyrazine	9.499	6.15	**13.72**	7.50	5.38	11.96
**Maltol**	**caramel**	**Alcohol**	10.672	7.25	8.37	**12.06**	10.05	9.05
4H-Pyran-4-one, 2,3-dihydro-3,5-dihydroxy-6-methyl-	caramel	Pyrans	11.839	0.00	0.00	0.00	0.00	**3.56**
Pyrazine, 3,5-diethyl-2-methyl-	green and nutty	Pyrazine	12.483	0.00	**6.14**	0.00	0.00	0.00

Table S2. Volatile compounds in roasted coffee beans (continued)

Compound	Aroma	Group	rt	Total peak area percentage (%)				
				OC	C	LP	PM+LP	PM
1H-Pyrrole, 1-(2-furanylmethyl)-	bread, roasted almond, popcorn, malt, roasted chicken, beer and sandalwood oil	Pyrrole	13.506	3.05	3.24	2.66	**4.29**	1.79
2-Furancarboxaldehyde, 5-(hydroxymethyl)-	warm-herbaceous, winey-ethereal, hungarian chamomile, tobacco	Furans	15.297	3.59	4.58	5.77	5.23	**6.62**
2-Cyclohexen-1-one, 4-(3-hydroxy-1-butenyl)-3,5,5-trimethyl-, [R-[R*,R*-(E)]]-	tobacco and tobacco smoke	Ketones	16.108	0.49	0.86	0.98	1.20	**1.21**
2-Isoamyl-6-methylpyrazine	sour	Pyrazine	16.232	0.00	**3.89**	1.97	0.00	1.21
10-Hydroxydecanoic acid	honey	Acid	17.357	0.00	0.00	**1.68**	0.00	0.00
Nonanoic acid	coconut	Acid	17.657	0.86	**0.92**	0.21	0.51	0.10
4-Vinylguaiacol	clove, spicy	Phenols	18.890	17.13	17.44	**18.57**	13.36	9.71
Limonen-6-ol, pivalate	sweet, orange, citrus	Terpene	19.305	0.00	0.00	**0.37**	0.00	0.13
Tetradecane, 2,6,10-trimethyl-	fatty, dairy, coconut	Esters	19.448	0.00	0.00	**0.37**	0.00	0.32
Geranyl isovalerate	floral, fruity, apple	Phenols	19.880	1.74	0.00	1.62	0.15	**2.15**

Table S2. Volatile compounds in roasted coffee beans (continued)

Compound	Aroma	Group	rt	Total peak area percentage (%)				
				OC	C	LP	PM+LP	PM
α-Furfuryliden-α-furylmethylamine	coffee-like, burnt aroma, sweet and spicy	Furans	22.963	1.51	2.15	**7.51**	6.82	5.74
5-Methyl-2-phenyl-2-hexenal	cocoa, chocolate	Aldehydes	26.058	0.00	0.62	**0.67**	0.00	0.58
Megastigmatrienone	tobacco	Phenols	29.451	0.13	0.13	**0.16**	0.13	0.00
Hexadecane	gasoline	Alkane	30.327	0.15	**0.24**	0.20	0.21	0.14

ordinary coffee (OC), control or non-fermentation (C; uninoculation), *P. motobuensis* JT-A29 (PM), *L. plantarum* JT-PN39 (LP) and mixed starter cultures (PM+LP) fermented coffee.
